# miR-26a-5p alleviates lipopolysaccharide-induced acute lung injury by targeting the connective tissue growth factor

**DOI:** 10.3892/mmr.2020.11643

**Published:** 2020-11-03

**Authors:** Hongyan Li, Tingting Yang, Zhaoxia Fei

**Affiliations:** 1 Department of Child Healthcare, Zibo Women & Children Hospital, Zibo, Shandong 255000, P.R. China; 2 General Internal Medicine, Qingdao Hospital of Traditional Chinese Medicine (Qingdao Hiser Hospital), Qingdao, Shandong 266033, P.R. China

**Keywords:** acute lung injury, lipopolysaccharide, microRNA-26a-5p, inflammation, apoptosis, connective tissue growth factor

## Abstract

The aim of the present study was to investigate the regulatory functions of microRNA (miR)-26a-5p on lipopolysaccharide (LPS)-induced acute lung injury (ALI) and its molecular mechanisms. The role of miR-26a-5p on an ALI mouse model was evaluated by examining the histological changes, wet/dry (W/D) ratio, myeloperoxidase (MPO) activity, malondialdehyde (MDA) expression levels in lung tissues and the survival of ALI mice. Moreover, the protein concentration and the number of neutrophils and lymphocytes in bronchoalveolar lavage fluid (BALF) was analyzed. To explore the effect of miR-26a-5p on inflammatory responses and apoptosis, the expression levels of tumour necrosis factor-α (TNF-α), interleukin (IL)-1β and IL-6 and apoptosis were measured by ELISA, terminal deoxynucleotidyl transferase-mediated dUTP nick end labelling staining and flow cytometry in BALF, A549 cells and lung tissues. B-cell lymphoma-2 (Bcl-2), Bax and cleaved caspase-3 in lung tissues were measured by western blotting and reverse transcription-quantitative PCR. Connective tissue growth factor (CTGF) was predicted as a direct target of miR-26a-5p using dual luciferase reporter assay. The present study sought to determine whether CTGF overexpression reversed the effect of miR-26a-5p on apoptosis and inflammatory responses in LPS-induced A549 cells. The data revealed that miR-26a-5p overexpression ameliorated LPS-induced ALI, which was implicated by fewer histopathological changes, W/D ratio, apoptosis in lung tissues and the survival of ALI mice. Moreover, miR-26a-5p overexpression alleviated LPS-induced inflammatory responses in ALI mice via the reduction of total protein, neutrophil and lymphocyte counts and the expression levels of TNF-α, IL-1β, IL-6, MDA and MPO activity in BALF. Similarly, miR-26a-5p overexpression decreased apoptosis and the expression of TNF-α, IL-1β and IL-6 in LPS-induced A549 cells. CTGF was a direct target of miR-26a-5p. CTGF overexpression reversed the effect of miR-26a-5p on cell apoptosis and inflammatory responses in LPS-induced A549 cells. The present study demonstrated that miR-26a-5p could attenuate lung inflammation and apoptosis in LPS-induced ALI by targeting CTGF.

## Introduction

Acute lung injury (ALI) is characterized by serious pulmonary inflammatory responses with a high incidence of morbidity and mortality (1). Some progress has been made in developing a therapeutic strategy and etiology, although the mortality rate of patients with ALI remains high (2). Consequently, there is a need to identify innovative therapeutic strategies and effective medications for the benefit of patients with ALI.

MicroRNAs (miRNAs; miRs) are a class of short non-protein coding RNAs with lengths of 19–25 nucleotides, which modulate target gene expression at the posttranscriptional level (3). An increasing number of studies have reported that miRNAs play critical roles in the progression of several lung diseases, including ALI (4–6). For example, Yang *et al* (5) reported that the upregulation of miR-140-5p protected mice from lipopolysaccharide (LPS)-induced ALI via the myeloid differentiation primary response 88 (MyD88)/NF-κB pathway by targeting Toll-like receptor (TLR)4. Xie *et al* (6) revealed that the downregulation of miR-34b-5p attenuated inflammatory responses and apoptosis in LPS-induced ALI of mice through targeting progranulin. However, the effect of miR-26a-5p on LPS-induced ALI and its related molecular mechanisms have not yet been reported.

Connective tissue growth factor (CTGF) is a multifunctional matricellular protein that belongs to the CTGF-Cyr61/Cef10-Nov (CCN) family. It was first reported in 1991 by Bradham *et al* (7). Increasing evidence has demonstrated that CTGF, a downstream effector of transforming growth factor-β1 (TGF-β1), can induce lung injury and contribute to pulmonary fibrosis (8–10). In addition, CTGF has been reported to have an important role in inflammatory diseases including rheumatoid arthritis, inflammatory kidney diseases and silicosis (11). Previous studies have suggested that miRNAs, such as miR-18a, regulates lung injury by modulating the expression of CTGF and the TGF-β1 pathway (12). However, whether miR-26a-5p exerts beneficial effects on the progression of LPS-induced ALI by modulating CTGF remains unclear.

The present research aimed to identify the potential effects of miRNA-26a-5p on LPS-induced ALI and its related molecular mechanisms *in vitro* and *in vivo*. These data confirmed that miR-26a-5p could attenuate lung inflammation and apoptosis in LPS-induced ALI by targeting CTGF. Thus, miR-26a-5p may serve as an important target for the treatment of ALI.

## Materials and methods

### 

#### Animals

A total of 230 male C57BL/6 mice (6–8 weeks; 20–25 g) were obtained from Beijing Vital River Laboratory Animal Technology Co., Ltd. All mice were housed in climate-controlled quarters with a 12-h light/dark cycle and provided water and standard laboratory chow *ad libitum*. The experimental procedures were performed in accordance with the Guide for the Care and Use of Laboratory Animals and approved by the Ethics committee of Zibo Women & Children Hospital.

#### Experimental protocol and animal models of ALI

The mice were intratracheally injected with 5 mg/kg LPS (Beijing Solabio Science & Technology C., Ltd.), dissolved in phosphate buffer saline (PBS). A total of 24 h prior to LPS treatment, the mice were administered with intraperitoneal injections of miR-26a-5p mimic (20 mg/kg) or miR-26a-5p mimic negative control (NC) (20 mg/kg) twice per day for 2 days consecutively. Subsequently, the mice were randomly divided into five groups (n=38/group): Control group (normal mice without any treatment), Sham group (received PBS), LPS group (received LPS), LPS + mimic negative control (NC) group (received LPS and miR-26a-5p mimic NC) and LPS + miR-26a-5p mimic group (received LPS and miR-26a-5p mimic). All mice were sacrificed by cervical dislocation under anesthesia via intraperitoneal injection of 50 mg/kg pentobarbital sodium at 24 h after LPS treatment for the subsequent experiment. The criteria used to confirm death were as follows: Cessation of heartbeat for more than 5 min and no pupillary reflex to strong light. The miR-26a-5p mimic and miR-26a-5p mimic NC were purchased from Takara Biotechnology Co., Ltd. The sequences used were as follows: miR-26a-5p mimic, 5′-UUCAAGUAAUCCAGGAUAGGCU-3′ (sense); 5′-CCUAUCCUGGAUUACUUGAAUU-3′ (antisense); and miR-26a-5p mimic NC, 5′-UUCUCCGAACGUGUCACGUTT-3′ (sense); 5′-ACGUGACACGUUCGGAGAATT-3′ (antisense).

In addition, survival experiments were performed in the four mice groups (n=10/group; Sham, LPS, LPS + mimic NC, LPS + miR-26a-5p mimic). The survival of mice was monitored for 84 h.

#### Lung wet/dry (W/D) weight ratio

Lung edema was evaluated according to the wet/dry weight ratio of lung tissues. After the mice (n=6) were sacrificed, the right lungs were excised and immediately weighed to obtain the wet weight. Subsequently, the lung samples were dried at 80°C for 48 h to obtain the dry weight (Lung wet/dry ratio=wet weight/dry weight).

#### Myeloperoxidase (MPO) and malondialdehyde (MDA) assay

After the mice (n=6) were sacrificed, the right lung tissues were collected and homogenized in 4-(2-hydroxyethyl)-1-piperazineethanesulfonic acid containing 0.5% cetyltrimethyl ammonium bromide. Then, the activity of MPO (cat. no. A044) and the content of MDA (cat. no. A003-1) were measured by using the corresponding test kits obtained from Nanjing Jiancheng Bioengineering Institute in accordance with the manufacturer's instructions.

#### Protein concentration and cell counts in bronchoalveolar lavage fluid (BALF)

After the mice (n=6) were sacrificed, the BALF was collected by injection and retraction of 1.5 ml PBS three times, and was centrifuged at 1,200 × g for 10 min at 4°C. Subsequently, the supernatants were collected for the assessment of total protein concentration by the bicinchoninic acid (BCA) method. The cell pellet was resuspended in PBS, and the neutrophil and lymphocyte counts were determined using a hemocytometer following Wright-Giemsa staining for 10 min at room temperature.

#### Hematoxylin and eosin (H&E) staining

After the mice (n=5) were sacrificed, the right lung tissues were collected and fixed in 4% formaldehyde at room temperature for 24 h. Then, the lung tissues were dehydrated in graded concentrations of ethanol, embedded in paraffin, and transversely cut into 5-µm thick sections. Finally, the histological changes in lung tissues were analyzed by H&E staining under an optical microscope (magnification, ×100). In addition, the histological score of the lung tissues was calculated by assessing the alveolar congestion, hemorrhage, inflammatory cell infiltration and alveolar wall thickness, and graded according to a five-point scale from 0 to 4 as follows: 0=no damage, l=mild damage, 2=moderate damage, 3=severe damage and 4=very severe damage (13).

#### Terminal deoxynucleotidyl transferase-mediated dUTP nick end labelling (TUNEL) staining

After the mice (n=5) were sacrificed, the apoptotic cells in the harvested lung tissues were detected by using the TUNEL staining kit supplied by Shanghai Ruisai Biotechnology Co., Ltd. following the manufacturer's protocol. Five random fields were viewed in each section and the rate of positive cells was analyzed under a fluorescence microscope at a magnification of ×400.

#### Cell culture and transfection

The human type II alveolar epithelial cells (A549), supplied by American Type Culture Collection, were maintained in Dulbecco's Modified Eagle's Medium (DMEM; Gibco; Thermo Fisher Scientific, Inc.), supplemented with 10% fetal bovine serum (FBS; Invitrogen; Thermo Fisher Scientific, Inc.) and 1% penicillin/streptomycin (Invitrogen; Thermo Fisher Scientific, Inc.) at 37°C in a humidified atmosphere with 5% CO_2_. The miR-26a-5p mimic, pcDNA3.1-CTGF and their negative controls were obtained from Takara Biotechnology Co., Ltd. Briefly, 10 nM miR-26a-5p mimic or pcDNA3.1-CTGF were transfected into A549 cells (6×10^6^) using Lipofectamine^®^ 3000 (Invitrogen; Thermo Fisher Scientific, Inc.), at room temperature for 48 h according to the manufacturer's protocol. The transfected A549 cells were randomly divided into the Control group (no treatment), LPS group (treated with LPS), LPS + mimic NC group (treated with LPS and miR-26a-5p mimic negative control), LPS + miR-26a-5p mimic group (treated with LPS and miR-26a-5p mimic), LPS + miR-26a-5p mimic + oe-CTGF NC group (treated with LPS, miR-26a-5p mimic and pcDNA3.1-CTGF NC) and LPS + miR-26a-5p mimic + oe-CTGF (treated with LPS, miR-26a-5p mimic and pcDNA3.1-CTGF). After transfection for 48 h, the A549 cells were treated with LPS (Sigma-Aldrich; Merck KGaA) for 24 h and analyzed.

#### Cell viability assay

To assess cell viability, 3-(4,5-dimethylthiazol-2-yl)-2,5-diphenyltetrazolium bromide (MTT) assay was used. The transfected A549 cells were seeded into 96-well plates at a density of 1×10^3^ cells/well. After 24 h of LPS stimulation, A549 cells were incubated with 20 µl of MTT at a final concentration of 5 mg/ml (Sigma-Aldrich; Merck KGaA) in the dark for 4 h. Subsequently, dimethyl sulfoxide (DMSO, 150 µl) was added to each well. Finally, the cell viability was determined by measuring absorbances at 490 nm by using a microplate reader.

#### Flow cytometry

The A549 cells undergoing apoptosis were detected with an Annexin V-fluorescein isothiocyanate (FITC) apoptosis detection kit (BD Biosciences) following the manufacturer's instructions. Briefly, 24 h after LPS stimulation, the A549 cells from different groups were harvested, washed with PBS and resuspended in Annexin-binding buffer. Propidium iodide (PI) and FITC-conjugated Annexin V were added into the cell suspensions and maintained in the dark for 15 min at 37°C. The apoptotic cells were then analyzed using a flow cytometer (BD FACScalibur; BD Biosciences).

#### Enzyme-linked immunosorbent assay (ELISA)

The levels of interleukin (IL)-1β, IL-6 and tumor necrosis factor (TNF)-α in mice BALF (n=6) and A549 cells were determined using an ELISA kit (BioLegend, Inc.) in accordance with the manufacturer's instructions. The ELISA kits for mice BALF were as follows: IL-1β (cat. no. 432601), IL-6 (cat. no. 431304) and TNF-α (cat. no. 430904). The ELISA kits for A549 cells were as follows: IL-1β (cat. no. 579409), IL-6 (cat. no. 430504) and TNF-α (cat. no. 430204).

#### Dual luciferase reporter gene assay

TargetScan (14) was used to predict the targeting relationship between miR-26a-5p and CTGF. The 3′-untranslated region (3′-UTR) of CTGF, with wild-type or mutant binding sites for miR-26a-5p, was amplified and cloned into the pGL3 luciferase vector (Promega Corporation) to generate the plasmid pGL3-WT-CTGF-3′-UTR (CTGF-WT) or pGL3-Mut-CTGF-3′-UTR (CTGF-MT). For the luciferase reporter assay, A549 cells were co-transfected with reporter vectors and miR-26a-5p mimics or miR-26a-5p mimics NC using Lipofectamine^®^ 3000 (Invitrogen; Thermo Fisher Scientific, Inc.). A total of 48 h after the transfection, luciferase activity was detected by using a dual luciferase kit (Promega Corporation). Firefly luciferase activity was normalized to *Renilla* luciferase activity.

#### RNA extraction and reverse transcription-quantitative PCR (RT-qPCR)

Lung tissue was collected after the mice (n=5) were sacrificed. Total RNA was extracted from A549 cells and lung tissues by TRIzol^®^ Reagent (Thermo Fisher Scientific, Inc.), based on the manufacturer's protocols. Total RNA (2 µg) was added to the reverse transcription reaction to synthesize complementary DNA (cDNA) using SuperScript™ IV First-Strand Synthesis System (Invitrogen; Thermo Fisher Scientific, Inc.). Thereafter, RT-qPCR was performed on a 7500 Real-time PCR System with MirVana™ qRT-PCR miRNA (Invitrogen; Thermo Fisher Scientific, Inc.). The PCR reaction conditions were as follows: 95°C for 10 min followed by 40 cycles at 95°C for 10 sec, 60°C for 20 sec, and 72°C for 30 sec. The primers used in the present study were as follows: miR-26a-5p forward (F), 5′-GGATCCGCAGAAACTCCAGAGA-3′ and reverse (R), 5′-TTGGAGGAAAGACGATTTCCGT-3′; U6 F, 5′-GCGCGTCGTGTAAAGCGTTC-3′ and R, 5′-GTGCAGGGTCCGAGGT-3′; CTGF F, 5′-CAAGGGCCTCTTCTGTGACT-3′ and R, 5′-ACGTGCACTGGTACTTGCAG-3′; B-cell lymphoma-2 (Bcl-2) F, 5′-ATGTGTGTGGAGAGCGTCAA-3′ and R, 5′-GCCGGTTCAGGTACTCAGTC-3′; Bax F: 5′-TCTGACGGCAACTTCAACTG-3′, R: 5′-GGAGGAAGTCCAATGTCCAG-3′; cleaved caspase-3 F: 5′-CTCGGTCTGGTACAGATGTCG-3′, R: 5′-TGGCTCAGAAGCACACAAAC-3′; GAPDH F, 5′-GCACCGTCAAGGCTGAGAAC-3′ and R, 5′-ATGGTGGTGAAGACGCCAGT-3′. For the normalization of the expression levels, U6 and GAPDH were used as internal controls. The relative expression levels were calculated using the 2^−ΔΔCq^ method (15).

#### Western blot analysis

Lung tissue was collected after the mice (n=5) were sacrificed. Total proteins were extracted from A549 cells and lung tissues by using lysis buffer (Beijing Solarbio Science & Technology Co., Ltd.) containing phenylmethylsulfonyl fluoride (PMSF). The concentration of proteins was measured with a BCA protein assay kit (Beyotime Institute of Biotechnology). Protein samples (50 µg) were loaded on 10% sodium dodecyl sulfate-polyacrylamide gel for electrophoresis and then transferred onto polyvinylidene difluoride (PVDF) membranes (Merck KGaA). After blocking with 5% skimmed milk for 2 h at room temperature, the membranes were incubated at 4°C overnight with the following primary antibodies supplied by Cell Signaling, Inc.: Bax (1:1,000; product no. 14796), cleaved caspase-3 (1:1,000; product no. 9661), Bcl-2 (1:1,000; product no. 3498), CTGF (1:1,000; product no. 86641) and GAPDH (1:1,000; product no. 5174). After washing with TBST (20% Tween-20) three times, the membranes were incubated with the horseradish peroxidase-conjugated anti-rabbit IgG secondary antibody (1:5,000; cat. no. 7040; Cell Signaling Technology, Inc.) at room temperature for 1 h. Finally, the protein bands were visualized with an ECL system (Thermo Fisher Scientific, Inc.) and then analyzed by using Image Lab™ Software (version 3.0; Bio-Rad Laboratories, Inc.).

#### Statistical analysis

The statistical analysis was performed using SPSS 23.0 software (IBM Corp.). Data in the present study are presented as the mean ± standard deviation (SD). Statistical differences were analyzed using the unpaired Student's t-test or one-way analysis of variance with Tukey's multiple comparison post-hoc test. Survival rates were assessed by the Kaplan-Meier method, and survival curves were compared by log-rank tests. P<0.05 was considered to indicate a statistically significant difference. All experimental data were obtained from at least three independent experiments.

## Results

### 

#### Overexpression of miR-26a-5p alleviates LPS-induced acute lung injury in mice

**Figure 1.**
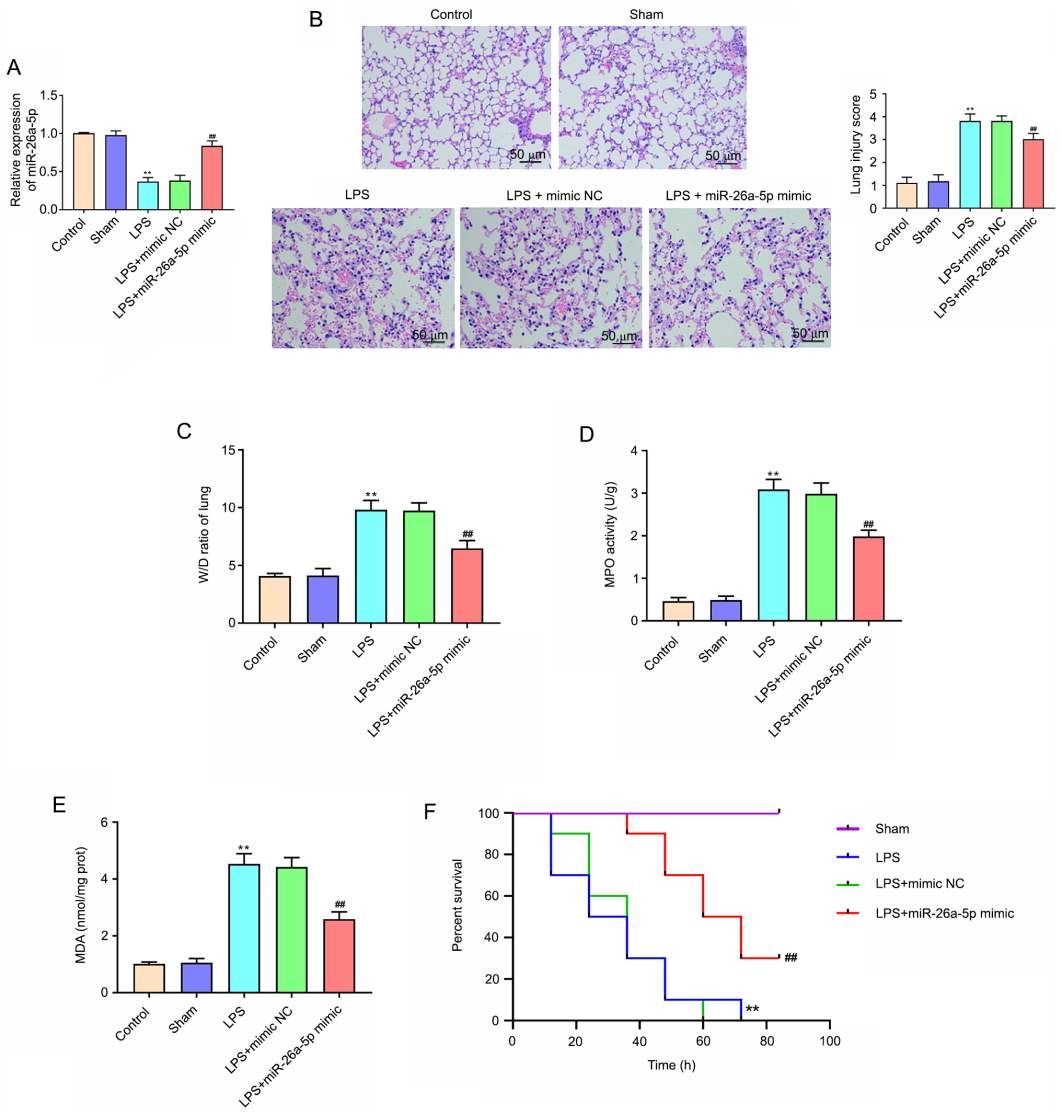
Overexpression of miR-26a-5p alleviates LPS-induced acute lung injury in mice. Mice in each group underwent intraperitoneal injection of miR-26a-5p mimics or NC mimics (20 mg/kg) 24 h before treatment with 5 mg/kg LPS. Mice were sacrificed after LPS administration for 24 h and then lung tissues were collected for analysis (except the survival experiment). (A) The expression of miR-26a-5p in lung tissues was detected by reverse transcription-quantitative PCR (n=5/group). (B) Pathological changes in the lung tissues observed by H&E staining (×100, magnification) (n=5/group). (C) The lung W/D weight ratio was assessed among the experimental groups (n=6/group). The activity of (D) MPO and the content of (E) MDA in lung tissues were detected using the corresponding test kits (n=6/group). (F) The survival rates were observed during 84 h following LPS treatment (n=10/group). Data were presented as the mean ± SD. **P<0.01 vs. Sham group; ^##^P<0.01 vs. LPS + mimic NC group. miR, microRNA; NC, negative control; LPS, lipopolysaccharide; W/D, weight/dry; MDA, malondialdehyde; MPO, myeloperoxidase.

The results of RT-qPCR revealed that the expression of miR-26a-5p was significantly decreased in the LPS group compared with the Sham group (P<0.01; Fig. 1A). Compared with the LPS + mimic NC group, the expression of miR-26a-5p was significantly increased in the LPS + miR-26a-5p mimic group (P<0.01; Fig. 1A). In addition, to explore the effect of miR-26a-5p on LPS-induced ALI, H&E staining and the W/D ratio of lung tissue were performed. As revealed in Fig. 1B, after the administration of LPS, the pulmonary edema, hemorrhage, alveolar wall thickening and inflammatory cell infiltration were markedly increased. Additionally, miR-26a-5p overexpression significantly ameliorated the histological changes induced by LPS (Fig. 1B). Lung injury scores also confirmed the result. Moreover, Fig. 1C revealed that the lung wet/dry weight ratio was significantly increased in the group treated with LPS compared with the Sham group (P<0.01). When compared with the LPS + mimic NC group, miR-26a-5p mimic significantly decreased the lung W/D ratio (P<0.01). As revealed in Fig. 1D and E, the activity of MPO and MDA content was significantly increased in LPS group compared with that of the Sham group (P<0.01). Correspondingly, upregulation of MPO and MDA was reversed by the overexpression of miR-26a-5p (P<0.01). In addition, the results revealed that LPS group mice survival rate was significantly reduced compared with the Sham group mice (P<0.01; Fig. 1F). Conversely, the mice survival rate in the LPS + miR-26a-5p mimic group was significantly longer than the LPS + mimic NC group (P<0.01; Fig. 1F). Collectively, the results indicated that the overexpression of miR-26a-5p could alleviate LPS-induced ALI in mice.

#### Overexpression of miR-26a-5p alleviates lung tissue inflammation in LPS-induced ALI mice

**Figure 2.**
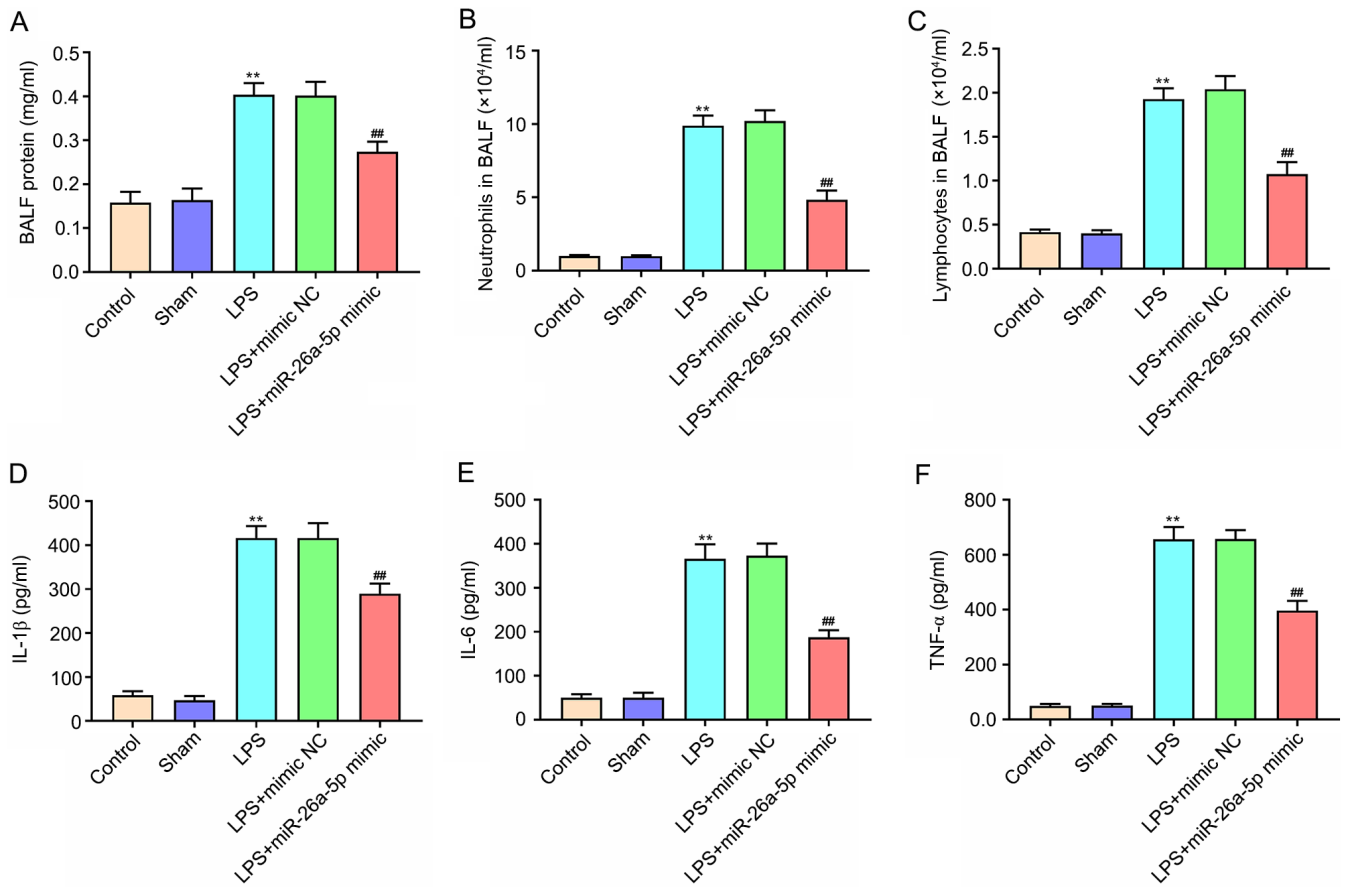
Overexpression of miR-26a-5p alleviates lung tissue inflammation in LPS-induced acute lung injury mice. Mice in each group underwent intraperitoneal injection of miR-26a-5p mimics or NC mimics (20 mg/kg) 24 h before treatment with 5 mg/kg LPS. And mice were sacrificed after LPS administration for 24 h and then BALF were collected for analysis. (A) The total protein concentration in BALF was measured by BCA method (n=6/group). The number of (B) neutrophils and (C) lymphocytes in BALF was determined using a hemocytometer (n=6/group). The expression level of (D) IL-1β, (E) IL-6 and (F) TNF-α in BALF was detected by ELISA (n=6/group). Data are presented as the mean ± SD. **P<0.01 vs. Sham group; ^##^P<0.01, vs. LPS + mimic NC group. miR, microRNA; NC, negative control; LPS, lipopolysaccharide; BALF, bronchoalveolar lavage fluid.

The protein expression levels and the number of neutrophils and lymphocytes in BALF were significantly increased in the LPS group compared with the Sham group (P<0.01; Fig. 2A-C). In contrast, miR-26a-5p overexpression significantly decreased the upregulation induced by LPS (P<0.01). The ELISA results confirmed that the expression of IL-1β, IL-6 and TNF-α were significantly increased after the administration of LPS (P<0.01; Fig. 2D-F). Furthermore, the upregulation of IL-1β, IL-6 and TNF-α was reversed by the overexpression of miR-26a-5p (P<0.01; Fig. 2D-F). These results revealed that the overexpression of miR-26a-5p could alleviate lung tissue inflammation in LPS-induced ALI in mice.

#### Overexpression of miR-26a-5p decreases lung tissue cell apoptosis in LPS-induced ALI mice

**Figure 3.**
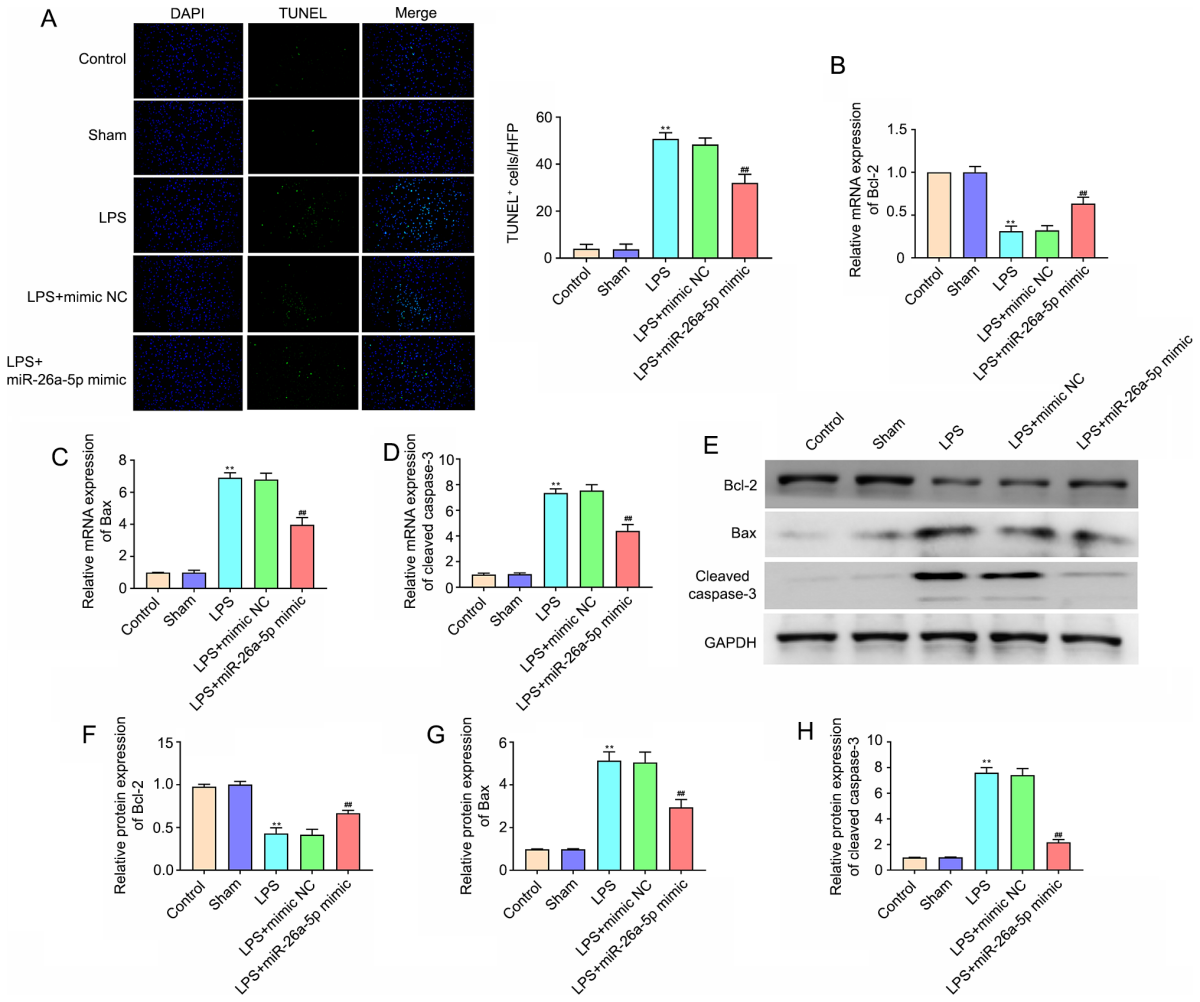
Overexpression of miR-26a-5p decreases lung tissue cell apoptosis in LPS-induced acute lung injury mice. Mice in each group were intraperitoneal injection of miR-26a-5p mimics or NC mimics (20 mg/kg) 24 h before treatment with 5 mg/kg LPS. And mice were sacrificed after LPS administration for 24 h and then lung tissues were collected for analysis. (A) Apoptotic cells in the lung tissues were detected by TUNEL staining (magnification, ×400) (n=5/group). The mRNA expression of (B) Bcl-2, (C) Bax and (D) cleaved caspase-3 in the lung tissues was detected by reverse transcription-quantitative PCR (n=5/group). (E) Representative western blot bands of Bcl-2, Bax and cleaved caspase-3. (F) Semi-quantitative data for the level of (F) Bcl-2, (G) Bax and (H) cleaved caspase-3 expression (n=5/group). Data were presented as the mean ± SD. **P<0.01 vs. Sham group; ^##^P<0.01 vs. LPS + mimic NC group. miR, microRNA; NC, negative control; LPS, lipopolysaccharide; TUNEL, Terminal deoxynucleotidyl transferase-mediated dUTP nick end labelling.

To evaluate the cell apoptosis in lung tissues, TUNEL staining was performed (Fig. 3A). The number of apoptotic cells in the lung tissues in the LPS group was significantly increased compared with that in the Sham group (P<0.01). miR-26a-5p overexpression also significantly reduced the LPS-induced apoptosis of lung cells (P<0.01). In addition, apoptosis-related proteins were detected via RT-qPCR and western blotting. The results of Fig. 3B-H demonstrated that the mRNA and protein expression levels of Bcl-2 in the LPS treatment group were lower than those in the Sham group (P<0.01), whereas the expression levels of Bax and cleaved caspase-3 were significantly higher (P<0.01). In addition, the overexpression of miR-26a-5p significantly reversed these effects on Bcl-2, Bax and cleaved caspase-3 (P<0.01). All data indicated that the overexpression of miR-26a-5p could decrease lung tissue cell apoptosis in LPS-induced ALI in mice.

#### Overexpression of miR-26a-5p decreases apoptosis and inflammatory responses in LPS-induced A549 cells

**Figure 4.**
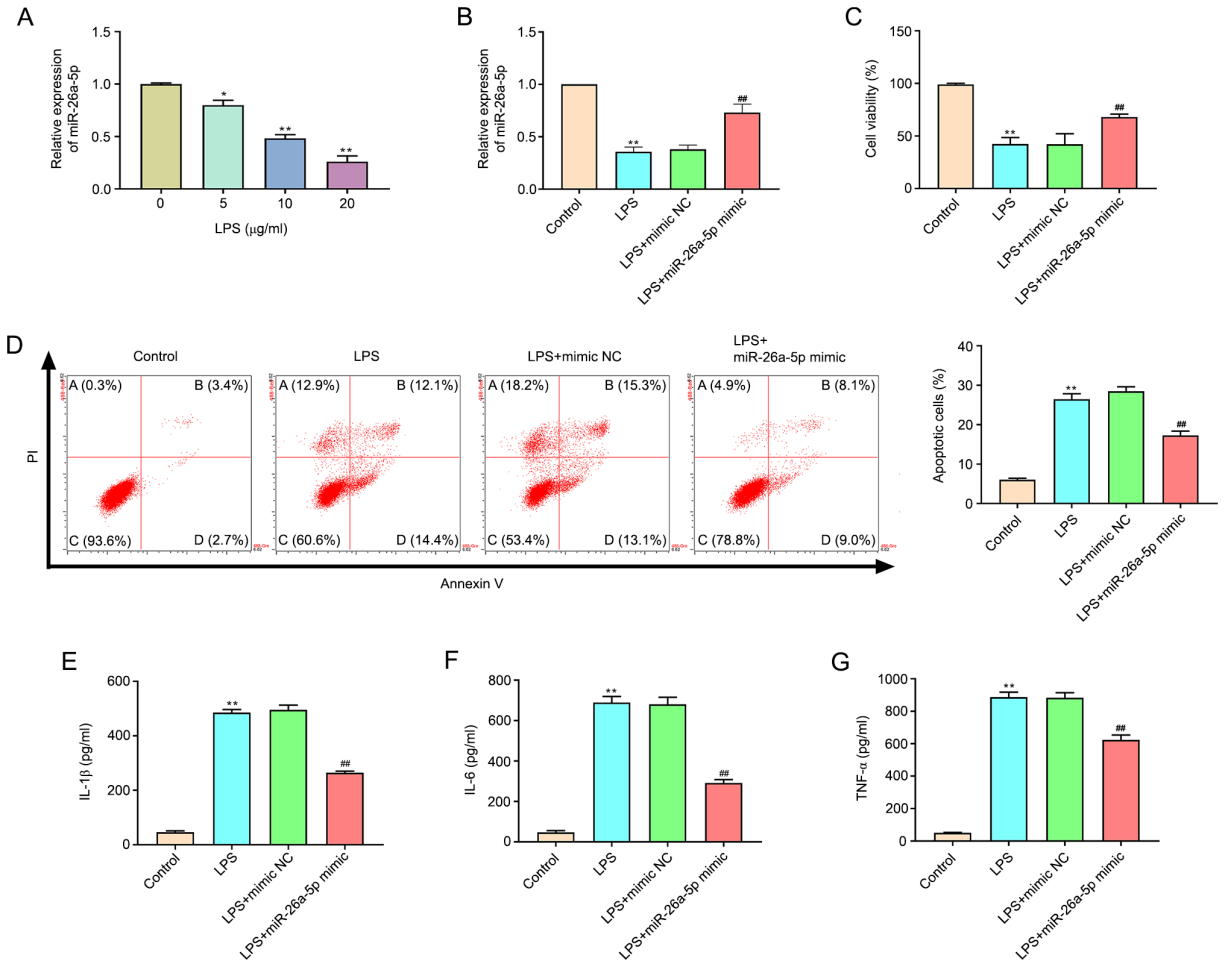
Overexpression of miR-26a-5p decreases apoptosis and inflammatory responses in LPS-induced A549 cells. A549 cells were transfected with miR-26a-5p mimic or mimic NC (10 nM), and then treated with LPS for 24 h, followed by the assessment of cell apoptosis and inflammatory response. (A) The expression of miR-26a-5p was detected by reverse transcription-quantitative PCR in A549 cells treated with various concentrations (0, 5, 10 and 20 µg/ml) of LPS. (B) The expression of miR-26a-5p was detected in transfected A549 cells. (C) The cell viability was measured by MTT assay in transfected A549 cells. (D) Apoptotic cells were detected by flow cytometry in transfected A549. The expression levels of (E) IL-1β, (F) IL-6 and (G) TNF-α were detected by ELISA in transfected A549 cells. Data were presented as the mean ± SD. (A) *P<0.05, **P<0.01 vs. LPS (0 µg/ml) group. (B-G) **P<0.01 vs. Control group; ^##^P<0.01 vs. LPS + mimic NC group. miR, microRNA; NC, negative control; LPS, lipopolysaccharide.

As revealed in Fig. 4A, the expression of miR-26a-5p in LPS-treated cells was significantly decreased compared with the untreated cells in a dose-dependent manner (P<0.05, P<0.01). A concentration of 20 µg/ml LPS was selected for subsequent experiments. Transfection efficacy was confirmed by the significantly augmented expression of miR-26a-5p at the mRNA expression level in A549 cells transfected with the miR-26a-5p mimic (Fig. 4B). MTT assay results confirmed that the administration of LPS significantly restricted cell viability (P<0.01; Fig. 4C). Correspondingly, miR-26a-5p overexpression significantly increased the cell viability of A549 cells relative to the LPS + mimic NC group (P<0.01), which indicated that miR-26a-5p overexpression increased the viability of LPS-induced A549 cells. In addition, the results of flow cytometry (Fig. 4D) revealed that the administration of LPS significantly promoted A549 cell apoptosis (P<0.01). However, the overexpression of miR-26a-5p significantly reduced the cell apoptosis induced by LPS (P<0.05). The present study also detected inflammatory cytokines in A549 cells and determined that the expression levels of IL-1β, IL-6 and TNF-α were significantly increased after LPS administration (P<0.01; Fig. 4E-G). The upregulation of IL-1β, IL-6 and TNF-α was significantly reversed by the overexpression of miR-26a-5p (P<0.01). Altogether, the present data indicated that the overexpression of miR-26a-5p could decrease apoptosis and inflammatory responses in LPS-induced A549 cells.

#### CTGF is the target gene of miR-26a-5p

**Figure 5.**
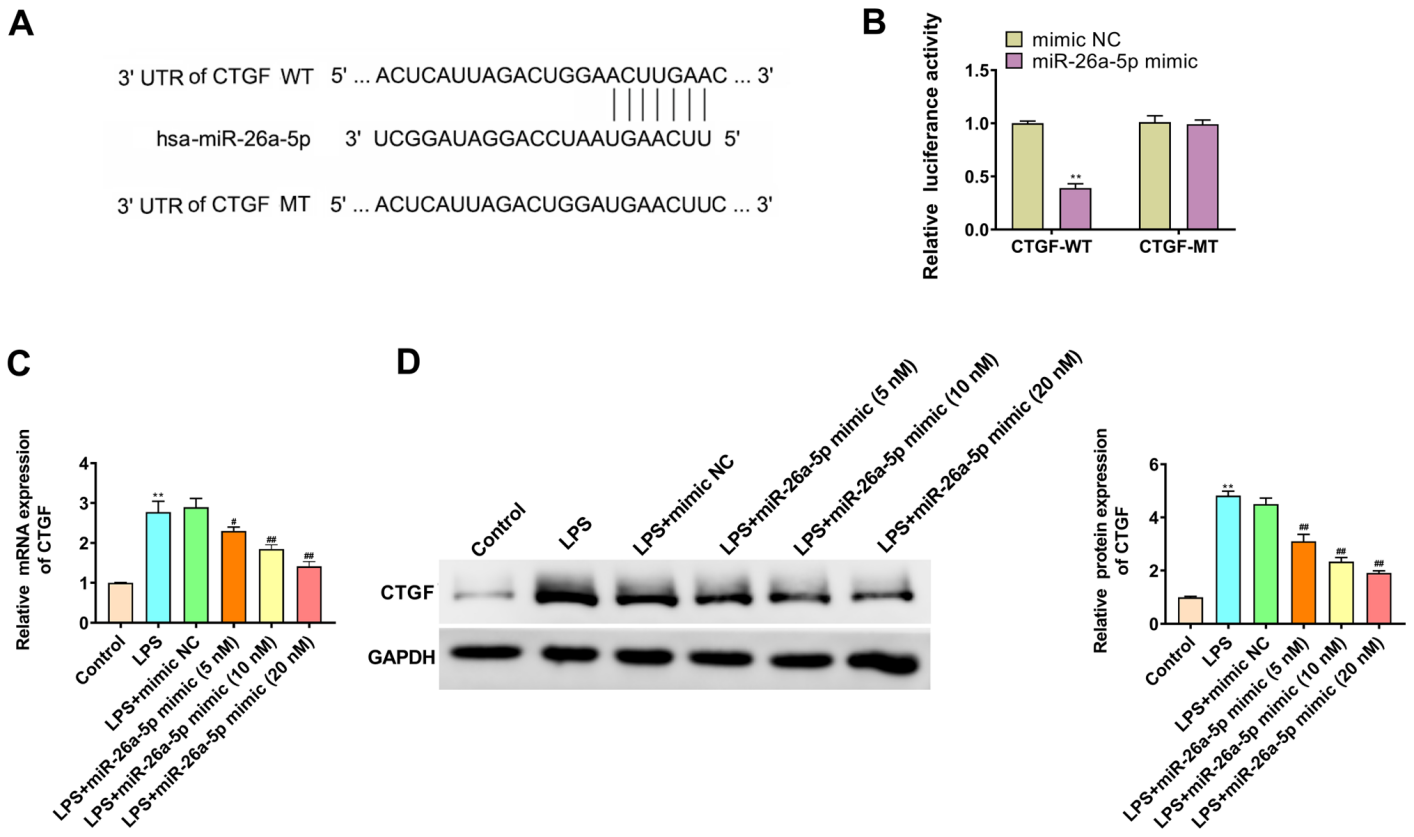
CTGF is the target gene of miR-26a-5p. (A) The binding target of miR-26a-5p and CTGF was predicted by TargetScan software 7.2. (B) Luciferase activity of CTGF-WT and CTGF-MT following the co-transfection of miR-26a-5p mimic or mimic NC in A549 cells detected by dual-luciferase reporter gene assay. (C) The mRNA expression of CTGF was detected by reverse transcription-quantitative PCR in A549 cells transfected with mimic NC or various concentrations (5, 10 and 20 nM) of miR-26a-5p. (D) The protein expression of CTGF was detected by western blotting in A549 cells transfected with mimic NC or various concentrations (5, 10 and 20 nM) of miR-26a-5p. Data were presented as the mean ± SD. (B) **P<0.01 vs. mimic NC group. (C and D) **P<0.01 vs. Control group; ^#^P<0.05, ^##^P<0.01 vs. LPS + mimic NC group. miR, microRNA; NC, negative control; LPS, lipopolysaccharide; CTGF, connective tissue growth factor; UTR, untranslated region; WT, wild-type; MT, mutant.

TargetScan predicted that the binding site of miR-26a-5p was the 3′-UTR region in CTGF (Fig. 5A). The dual luciferase reporter gene assay results revealed that the overexpression of miR-26a-5p significantly decreased the luciferase activity in the CTGF-WT group (P<0.01; Fig. 5B); however, it had no significant effect on the luciferase activity in CTGF-MT group (P>0.05; Fig. 5B). Furthermore, the study further elucidated whether miR-26a-5p negatively regulated CTGF in A549 cells by RT-qPCR (Fig. 5C) and western blotting (Fig. 5D). The results revealed that the mRNA and protein expression levels of CTGF in the LPS group were higher than those in the Control group (P<0.01). miR-26a-5p also significantly reversed the effect of LPS on CTGF in a dose-dependent manner. These results indicated that CTGF was a target gene of miR-26a-5p.

#### miR-26a-5p inhibits apoptosis and inflammatory responses via targeting CTGF in A549 cells

**Figure 6.**
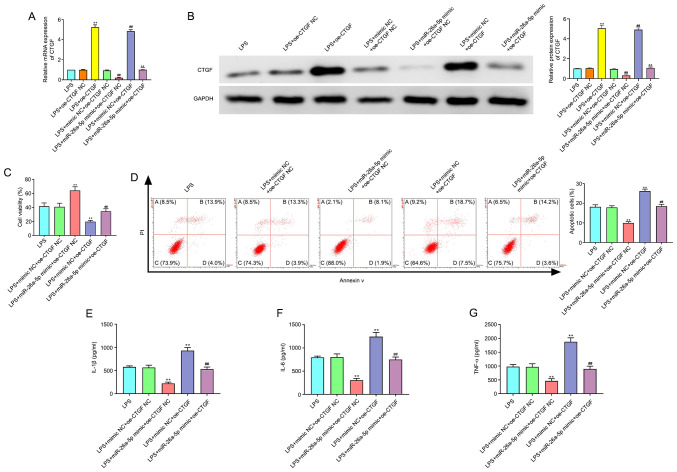
CTGF overexpression reverses the effect of miR-26a-5p on the apoptosis and inflammatory responses in LPS-induced A549 cells. A549 cells were co-transfected with miR-26a-5p mimic (10 nM), oe-CTGF (10 nM), mimic NC (10 nM) and oe-CTGF NC (10 nM), and then treated with LPS for 24 h, followed by the assessment of cell apoptosis and inflammatory response. (A) The mRNA expression of CTGF was detected by reverse transcription-quantitative PCR in transfected A549 cells. (B) The protein expression of CTGF was measured by western blotting in transfected A549 cells. (C) Cell viability was detected by MTT assay in transfected A549 cells. (D) The cell apoptosis was detected by flow cytometry in transfected A549 cells. The expression levels of (E) IL-1β, (F) IL-6 and (G) TNF-α were detected by ELISA in transfected A549 cells. Data were presented as the mean ± SD. (A and B) **P<0.01 vs. LPS + oe-CTGF NC group; ^##^P<0.01 vs. LPS + mimic NC + oe-CTGF NC group; ^&&^P<0.01 vs. LPS + miR-26a-5p mimic + oe-CTGF NC or LPS + mimic NC + oe-CTGF group. (C-G) **P<0.01 vs. LPS + mimic NC + oe-CTGF NC group; ^##^P<0.01 vs. LPS + miR-26a-5p mimic + oe-CTGF NC or LPS + mimic NC + oe-CTGF group. miR, microRNA; NC, negative control; LPS, lipopolysaccharide; CTGF, connective tissue growth factor; oe, overexpression plasmid.

As revealed in Fig. 6A and B, the transfection efficacy of pcDNA3.1-CTGF, the CTGF overexpression plasmid, was confirmed by the significantly enhanced expression of CTGF at the mRNA and protein expression levels in A549 cells. In addition, the results of Fig. 6C-G revealed that CTGF overexpression significantly increased cell apoptosis and the expression levels of IL-1β, IL-6 and TNF-α, and decreased cell viability (P<0.01). Moreover, the overexpression of CTGF significantly reversed the effects of miR-26a-5p mimic on cell viability, apoptosis and inflammatory cytokines in LPS-induced A549 cells (P<0.01). Collectively, this data indicated that miR-26a-5p could inhibit apoptosis and inflammatory responses via targeting CTGF in A549 cells.

## Discussion

ALI is a serious lung disease that is often accompanied by acute inflammatory responses and can lead to respiratory failure (16). At present, the five-year survival rate of patients with ALI is reported to be lower than 50% (17). Therefore, further studies focusing on the development of novel therapeutic targets for the treatment of ALI are required. This research has confirmed that miR-26a-5p could attenuate lung inflammation and apoptosis in LPS-induced ALI by targeting CTGF.

Recent studies have reported that miRNAs, as gene expression switches, have an important role in the progression of ALI (18,19). A recent study has indicated that miR-802 was expressed at a low level in LPS-induced ALI and alleviated LPS-induced ALI by targeting Peli2 (20). Another recent study has revealed that miR-539-5p was underexpressed in sepsis-induced ALI and could protect mice from ALI (21). In the present study, the expression of miR-26a-5p was downregulated in LPS-induced ALI models *in vivo* and *in vitro*. It should be noted that miR-26a-5p has an important role in several injury models. For instance, Wei *et al* (22) have reported that miR-26a-5p overexpression could improve cerebral ischemia/reperfusion injury via attenuating cell apoptosis. Xing *et al* (23) have indicated that miR-26a-5p could protect against myocardial ischemia/reperfusion injury by modulating the PTEN/PI3K/AKT pathway. However, the function of miR-26a-5p in LPS-induced ALI is unclear. The present data revealed that the overexpression of miR-26a-5p markedly improved LPS-induced ALI by reducing the histopathological changes and W/D ratio in lung tissues, and thereby helped to improve the survival of ALI mice.

Previous studies have indicated that ALI is featured with the production of inflammatory factors, inflammatory cell infiltration and pulmonary epithelial cell apoptosis (24,25). Evidence from several clinical studies has indicated that TNF-α, IL-1β and IL-6 are the main inflammatory cytokines actively secreted in response to the inflammatory cascade in ALI (21,26). Previous studies have indicated that the excess inflammatory cytokines, such as TNF-α, IL-6 and IL-1β, ultimately cause cell death via apoptosis and pyroptosis (27). In addition, an increasing number of studies have demonstrated that miRNAs could exert a protective effect against LPS-induced ALI by inhibiting lung inflammation and pulmonary epithelial cell apoptosis. For example, miR-27a could alleviate lung inflammation and apoptosis in LPS-induced ALI mice by modulating the TLR4/MyD88/NF-κB pathway (28). Suo *et al* (29) have confirmed that miR-1246 could suppress ALI-induced inflammation and apoptosis by modulating NF-κB and Wnt/β-catenin pathways. In addition, miR-26a-5p is considered to be associated with inflammation and apoptosis in numerous diseases. For example, the overexpression of miR-26a-5p could markedly suppress neuropathic pain and neuroinflammation in rats with chronic sciatic nerve injury (30). Wen *et al* (31) have suggested that miR-26a-5p significantly decreased myocardial cell apoptosis and expression of inflammatory factors in acute myocardial infarction by targeting ADAM17. The present study also demonstrated that the overexpression of miR-26a-5p significantly decreased LPS-induced apoptosis and inflammatory factor expression levels in both ALI mice and cell models, which suggests that miR-26a-5p could exert a protective effect against LPS-induced ALI by inhibiting apoptosis and inflammatory responses.

CTGF, also referred to as CCN family protein 2 (CCN2) and one of six members of cysteine-rich, secreted, heparin-binding proteins with a modular structure, has an important role in tissue remodeling and fibrosis (32). Increasingly, research has indicated that CTGF is a downstream mediator of TGF-β1 that induces connective tissue cell proliferation and extracellular matrix deposition (8). The overexpression of CTGF has been observed in numerous fibrotic tissues, including kidney, lung, heart and liver, whereas in normal adult tissues or cells expression is at low or even undetectable levels (33). The present data also revealed that the expression of CTGF was significantly upregulated in LPS-induced A549 cells. Previous studies have indicated that inhibiting the upregulation of CTGF could attenuate bleomycin-induced ALI and pulmonary fibrosis (34). Furthermore, previous studies have reported that anti-CTGF therapy with neutralizing CTGF monoclonal antibodies could improve bleomycin-induced or radiation-induced lung fibrosis (9,35). Evidence from several studies has suggested that miRNAs, such as miR-18a and miR-26a, could modulate lung injury and lung fibrosis via the regulation of CTGF (12,36). Furthermore, a study by Li *et al* (37) has revealed that miR-26a-5p could alleviate pneumonia by targeting CTGF. The present data also confirmed that miR-26a-5p could negatively modulate the expression of CTGF. In addition, the present results also revealed that CTGF overexpression significantly reversed the effect of miR-26a-5p on cell apoptosis and inflammatory cytokines in LPS-induced A549 cells. Collectively, these results indicated that miR-26a-5p could inhibit apoptosis and inflammatory responses via targeting CTGF in LPS-induced ALI.

In summary, the present study confirmed that the expression of miR-26a-5p was downregulated in LPS-induced ALI mice and in A549 cells, while CTGF expression was upregulated. In addition, miR-26a-5p was demonstrated to attenuate lung inflammation and apoptosis in LPS-induced ALI by targeting CTGF. The findings of the present study provide new perspectives on miRNA-based diagnostic approaches against LPS-induced ALI. A key limitation of the present study was that only the effect of miR-26a-5p on CTGF expression was assessed; therefore, further investigation is required to verify the protective effect of miR-26a-5p against LPS-induced ALI.

## Data Availability

The datasets used and analyzed during the current study are available from the corresponding author on reasonable request.
